# Adoption of Soil and Water Conservation Practices and Farmer Perceptions of Soil Erosion: Implications for Sustainable Agriculture in Soro District, Central Ethiopia

**DOI:** 10.1155/tswj/5192374

**Published:** 2025-09-19

**Authors:** Esiyas Estefanos, Belayneh Bufebo, Tamene Betebo

**Affiliations:** ^1^Department of Rural Development and Agricultural Extension, College of Agricultural Sciences, Wachemo University, Hosanna, Ethiopia; ^2^Department of Natural Resources Management, College of Agricultural Sciences, Wachemo University, Hosanna, Ethiopia

**Keywords:** adoption, perception, probit regression model, soil erosion, Soro

## Abstract

Soil erosion remains a critical environmental and agricultural challenge in Ethiopia, particularly in highland areas such as the Soro District of Central Ethiopia, where land degradation directly threatens agricultural productivity and rural livelihoods. This study was undertaken to better understand the key factors influencing smallholder farmers' adoption of soil and water conservation practices, along with their perceptions of soil erosion and its impacts. The study employed a mixed methods approach, combining household surveys, focus group discussions, and field observations to gather both quantitative and qualitative data from a representative sample of smallholder farmers. Descriptive analysis, principal component analysis, and probit regression models were used to analyze the collected data. Probit regression analysis revealed that factors such as sex, education, size of farmland, interactions with extension services, participation in conservation training, and household income all played a significant and positive role in encouraging the adoption of soil and water management practices. In contrast, soil fertility was found to have a negative effect on the uptake of soil and water conservation techniques. Farmers perceived damaged conservation structures, soil loss, and farmland fragmentation as key consequences of severe soil erosion. While most farmers recognize soil erosion as a major threat to their land, adoption rates of sustainable practices remain uneven due to economic, institutional, and knowledge-based constraints. The study underscores the importance of integrating local perceptions into policy frameworks and promoting participatory approaches to enhance the uptake of conservation practices. These insights contribute to the design of more effective and context-specific land management strategies aimed at ensuring long-term environmental sustainability and agricultural resilience in the region.

## 1. Introduction

Soil erosion represents a significant environmental threat with extensive implications for agricultural sustainability and ecosystem health. The process entails the displacement of the uppermost soil layer, which contains the highest concentration of organic matter and essential nutrients. The loss of this fertile layer compromises soil productivity and diminishes the agricultural potential of affected lands. As topsoil erodes, crop yields decline, thereby posing a serious risk to global food production systems [1]. To ensure long-term agricultural viability and food security, the adoption of effective soil management strategies is imperative. Moreover, soil erosion disrupts natural hydrological processes, impeding groundwater recharge and reducing water availability for vegetation and ecosystems. The removal of key macronutrients including nitrogen, phosphorus, and potassium further constrains plant growth and undermines the integrity of terrestrial ecosystems [2].

Water-induced soil erosion remains a primary driver of land degradation and food insecurity, especially in developing nations. Since 1945, approximately 1.2 billion hectares of arable land have been adversely impacted by erosion, with an estimated 80% of this degradation occurring in low- and middle-income countries [3]. Such degradation poses serious threats to both current agricultural productivity and the long-term sustainability of food systems. An estimated 9 million hectares globally are considered severely degraded, significantly impairing their ecological functionality. The widespread nature of soil degradation is closely linked to unsustainable land use practices. Notably, only about 20% of global croplands have adopted conservation agriculture techniques, leaving the remaining 80% highly vulnerable to continued degradation [2].

The severity of soil erosion is particularly notable across sub-Saharan Africa, with Ethiopia exemplifying one of the most affected regions. The African continent loses between 5 and 6 million hectares of soil annually to erosion [4], and it hosts nearly 50% of the global population affected by this phenomenon [5]. In Ethiopia's highland regions, soil erosion is particularly severe: About 50% of the area is affected, with 25% classified as highly degraded and 4% as irreversibly damaged [6, 7].

Multiple anthropogenic and environmental factors contribute to soil erosion, including cultivation on steep slopes, insufficient recycling of crop residues, deforestation, overgrazing, and inadequate implementation of soil and water conservation (SWC) practices [8]. The cumulative effect of these drivers reduces soil productivity, exacerbates household food insecurity, and deepens rural poverty [9]. In Ethiopia, the ecological and economic impacts of severe soil erosion are profound, stemming from decades of unsustainable resource exploitation [10].

Soil erosion continues to pose a significant environmental and agricultural challenge in Ethiopia, particularly in highland regions such as the Soro District of Central Ethiopia. In this area, land degradation directly undermines agricultural productivity and threatens rural livelihoods. High population density has led to continuous cultivation, which exacerbates soil erosion and depletes soil fertility (SF), ultimately reducing crop yields [11]. Data from the district's Agriculture and Natural Resource Development Office indicate that approximately 40% of the district's land area is affected by erosion, contributing to the area's classification as one of the most chronically food-insecure regions.

The undulating topography of the Soro District further intensifies erosion, thereby limiting agricultural productivity. In response, a variety of SWC measures have been introduced throughout the district [12]. These interventions include SWC techniques, regulated livestock grazing, crop rotation, use of crop residues, and agroforestry practices (integrating trees into farming systems). Despite these initiatives, the overall adoption of SWC strategies as a long-term sustainable land management approach has remained low. As a result, expected improvements in land quality and productivity have not been realized. In fact, erosion has continued to intensify due to ongoing land use and insufficient maintenance of previously established conservation structures [13–16].

The limited adoption and sustainability of conservation practices reveal a significant knowledge gap concerning the underlying factors that influence their acceptance in the study area. In the Soro District, the drivers and barriers to implementing SWC measures remain insufficiently understood. As such, it is necessary to investigate why some smallholder farmers adopt these technologies, while others either adopt them partially or not at all. Understanding the interplay of farm-specific conditions and farmer-related characteristics is essential for identifying the key determinants of adoption behavior [17].

This study was undertaken to address these gaps by identifying the primary factors affecting the adoption of SWC technologies among smallholder farmers. It also examines farmer perceptions of soil erosion and its implications for sustainable agriculture and land management in the district. Beyond contributing to the academic understanding of these issues, this study is aimed at generating actionable insights for policy development, extension service (ES) design, research agendas, and development programming both within Soro and in other agroecological zones across Ethiopia. Unlike many previous studies, which have often been limited in scope and relied on narrowly defined statistical models focusing on either perception or adoption, this research applies a comprehensive methodological framework and rigorous statistical analysis. It utilizes multivariate probit regression, principal component analysis (PCA), and descriptive statistics to offer a nuanced and integrated analysis of both the perceptions and adoption behaviors of smallholder farmers. These findings provide a more holistic perspective on the challenges and opportunities associated with promoting sustainable land management practices in Ethiopia.

## 2. Methodology

### 2.1. Description of the Study Area

**Figure 1:**
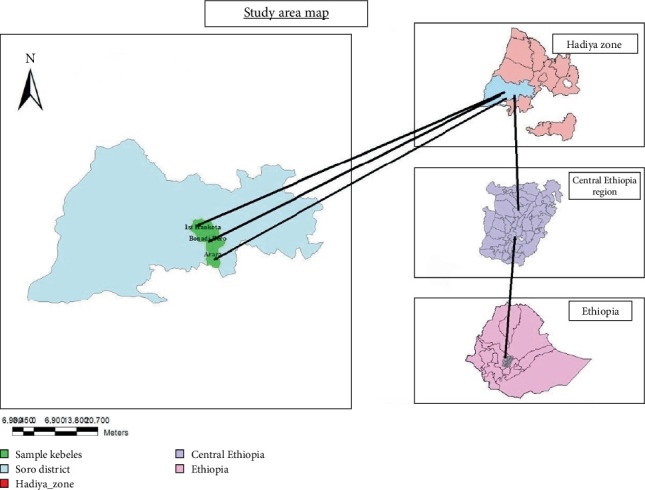
Map of the study area.

Soro District is located in the Central Ethiopia region. It is part of the Hadiya zone and situated at 7° 30⁣′–7° 43⁣′ north latitude and 37° 35⁣′–38° 05⁣′ east longitude (Figure 1). The district shares borders with the Kembata zone to the east, Mirab Soro to the southwest, the Omo River (which separates it from the Oromia region) to the west, Gombora to the north, Lemo to the northeast, and Duna to the southeast. The administrative center of Soro District is Gimbichu. Geographically, Soro District lies in the southwest direction from Addis Ababa (the capital city of Ethiopia), approximately 264 km away. The topography of the district consists of plains (31%), mountains (11%), and moderately sloping and steep lands (58%). The altitude ranges from 840 to 2850 m above sea level [18]. The mapping data were retrieved from the USGS GloVis website (http://glovis.usgs.gov/).

### 2.2. Climate

It falls within the moist *Woina-dega* agroecological zone, with 8% highland, 55% midland, and 37% lowland areas. The mean annual total rainfall is approximately 1260 mm, and the average temperature is 19°C [18]. The district experiences two rainy seasons: *Belg* (short rainy season from March to May) and *Kiremt* (longest rainy season from June to September). The highest rainfall occurs in July and August. Water resources include three main streams: Lintala (originating from Mount Shonkolla), Ajacho, and Gamunna, along with permanent springs for drinking water.

### 2.3. Land Use and Vegetation

The Soro District was once covered by thick forests, but increasing population pressure and agricultural expansion have significantly reduced its natural cover. In the place of indigenous vegetation, nonnative species such as *Eucalyptus globulus* and *Eucalyptus camaldulensis* now dominate. The current land use in Soro Woreda is divided as follows: Annual crops occupy 64.9%, perennial crops 5.58%, grazing areas 4.4%, bush and forest land 8.9%, degraded land 7.62%, and miscellaneous uses account for 8.6% [18].

### 2.4. Soil Characteristics

The area exhibits diverse topography and vegetation cover, resulting in variations in soil types. Dominant soil types include red-brown to red clayey soils on undulating and steep lands, as well as grayish soils on flat to undulating areas. Soil erosion is a concern, affecting productivity. Soil management depends on factors like soil type, fertility, slope, workability, water holding capacity, and erosion susceptibility [19].

### 2.5. Agriculture

Agriculture is the primary economic activity, combining traditional and mixed farming. Major crops cultivated include teff, maize, wheat, sorghum, haricot beans, enset, coffee, and various vegetables. Livestock rearing involves cattle, goats, sheep, donkeys, horses, and mules [19].

### 2.6. Research Design

The study used a cross-sectional research design to assess existing SWC practices, determinants of adoption, and farmers' perceptions of soil erosion causes and consequences. An explanatory research design was employed to explore cause-and-effect relationships between independent and dependent variables. Both quantitative and qualitative data were collected, with qualitative data obtained from focus group discussions (FGDs) and quantitative data from semistructured interviews.

### 2.7. Target Population and Sample Frame

The target population consisted of households residing in three *kebeles*: Bona, First Hankota, and Arera. *Kebeles* are the lowest administrative units. The total number of households in the study area was 1174.

### 2.8. Sample Size Determination

Yamane's sample size determination formula with a 95% confidence level and a 5% level of precision was applied to determine the sample size for this study. Suggested by [20], the sample size determination formula is given as

$$\begin{array}{cc} \; & n=\frac{N}{1+N\left(e\right)2\;},\\ n=\frac{1174}{1+1174{\left(0.05\right)}_{2}}=298.3=298 \end{array}$$

where *n* is the sample size, *N* is the population size of selected *kebeles* (lowest level of administration) which is (1174) households, and *e* is the level of precision, assumed as *e* = 5%. When this is applied to Equation (1), it gives (298.3~298). Therefore, the sample size for this specific study was 298 households (Table 1). After determining the total sample size of the selected three *kebeles*, the sample size of each selected *kebele* was then determined proportionally.

### 2.9. Sampling Techniques

Multistage sampling technique was employed to select the sample farm households. In the first stage, three kebeles (Bona, First Hankota, and Arera) were purposively selected based on existing SWC measures and the severity of soil erosion [21]. In the second stage, households within the selected kebeles were stratified into two groups: adopters (those practicing SWC) and nonadopters. The third stage involved systematic random sampling of 298 sample farmers from the overall population. The selection was proportional to the size of each kebele. Household lists were obtained from the kebele agricultural development offices. To select sample respondents from the total population, systematic sampling technique was adopted as follows: *N*/*n* = *k*, that is, *N* = total population, *n* = sample size that was conducted, and *k* = the first respondent position. Then, total sample follows the *k*-th respondent until selecting the total sample size. *k* = *N*/*n*: *k* = 1174/298 = 3.9. Therefore, every fourth respondent was taken.

### 2.10. Method of Data Collection

The study employed semistructured interviews to collect primary quantitative data from sampled households. Enumerators collected necessary information and conducted formal surveys using a predefined interview schedule. Two enumerators, well-versed in the local farming system and culture, collected data after receiving orientation and training. After the household interviews, a FGD was held to collect supplementary qualitative information on adoption constraints and the perception of smallholder farmers on causes, extent, and consequences of soil erosion. Three FGDs were used to gather qualitative data. A total of 27 participants (nine in each group) engaged in discussions. FGDs were organized considering sex and age composition. Participants shared their opinions, views, feelings, and perspectives on SWC practices, adoption factors, and perceptions of soil erosion. Sileshi et al. [22] describe the target population as the specific group relevant to a research study.

### 2.11. Method of Data Analysis

#### 2.11.1. Descriptive Analysis

Qualitative data were narratively summarized and described. For quantitative analysis: Descriptive statistics (frequency, percentage, mean, and standard deviation) were used. Inferential statistics (chi-square and *t*-tests) tested associations and mean differences.

#### 2.11.2. Analysis of Determinants Affecting Farmers' Adoption Decisions Regarding SWC Practices

The probit model was used to identify factors influencing farmers' adoption decisions. Previous studies have related farmers' technology adoption to various socioeconomic factors. The probit model approximates the cumulative normal distribution and simplifies estimation. Based on model specification [23], the probit model for determinants of adoption decisions is given as

(1) $$\begin{array}{c} \text{di}*=\propto 1\;{\text{x}}^{*}1\text{i}+\text{ui} \end{array}$$

where ui ~ *N*(0, 1)

 $$\begin{array}{c} \left\{\begin{array}{c} 0\text{ if di}*\leq 0 \end{array}\right. \end{array}$$

where *di*∗ is a latent variable that takes the value 1 if the farmer adopts and 0 otherwise, *x*∗1*i* is a vector of individual characteristics, and ∝1 is a vector of parameter to be estimated.

#### 2.11.3. Definition of Variables and Working Hypothesis

*Dependent variable*: The dependent variable is the adoption of soil and water conservation practices (ASWCP). It is treated as a dichotomous variable: 1 for households that adopted indigenous, introduced, or both practices on their farms during the survey and 0 for households that did not adopt such practices.

*Independent variables*: The study considers potential explanatory variables associated with SWC adoption among farmers.

*Age of the household head* (*AGE*): Continuous variable measured in years. Age can influence confidence in adopting technologies [24]. Previous research suggests that age significantly affects the ASWCP. Hypothesis: Age and adoption of conservation structures are negatively correlated (Table 2).

*Sex of the household head* (*SEX*): A dummy variable (1 for male household head, 0 for female). Sex influences access to information and perception of soil erosion [24]. Female-headed households tend to adopt conservation practices less frequently than male-headed households. Hypothesis: Positive relation between sex and ASWCP (Table 2).

*Household size* (*HS*): It is a continuous variable measured in man equivalents. A larger active labor force may invest more in improved conservation measures [25]. Larger families find it easier to build physical SWC structures. Hypothesis: Larger family size positively affects the ASWCP (Table 2).

*Education level* (*EDU*): Continuous variable measured in years of schooling completed. Higher EDU positively influence adoption [26]. Households with more educated heads tend to adopt conservation practices. Hypothesis: EDU correlates positively with adoption.

*Farm size* (*FS*): Continuous variable measured by the area of the plot (hectares) owned by farmer households. Empirical studies show a positive effect of FS on conservation adoption. Larger FS tolerate the risk of losing cultivation land due to conservation structures [27]. Hypothesis: FS positively affects SWC adoption (Table 2).

*Livestock ownership* (*LIO*): Continuous variable measured in tropical livestock units (TLUs). LIO influences adoption differently across areas. Generally, livestock holding contributes positively to adopting agricultural technologies [28]. Hypothesis: LIO positively affects adoption of conservation practices (Table 2).

*Land ownership* (*LAO*): A dummy variable (1 if farmland is owned by the farmer, 0 otherwise). LAO directly impacts conservation decisions [26]. Previous research highlights its significance. Hypothesis: LAO positively influences farmers' adoption of conservation practices (Table 2).

*ES*: A dummy variable (1 if the farmer received ES, 0 otherwise). Access to ES significantly affects the ASWCP [29]. Previous studies show a positive relation between ES and conservation adoption. Hypothesis: ES positively affects ASWCP (Table 2).

*Training on SWC* (*TSWC*): A dummy variable (1 if the farmer participated in SWC trainings, 0 otherwise). Training participation significantly influences adoption decisions [26]. Access to training positively influences adopting soil conservation practices. Hypothesis: Training has a positive influence on adoption (Table 2).

*Household total income* (*HTI*): Continuous variable measured in birr (income from both farm and off-farm activities). Total income significantly affects adoption decisions. Households with higher total income tend to adopt SWC practices [30]. Hypothesis: Total income positively relates to physical conservation adoption (Table 2).

*Off-farm income* (*OFI*): Measured in birr (income from activities outside the farm). Participation in off-farm activities influences conservation adoption [27]. Hypothesis: Off-farm income negatively affects the adoption of conservation practices.

*SF*: A dummy variable (1 for fertile farmland, 0 otherwise). SF impacts adoption decisions. There is a negative correlation between SF and conservation investment [10]. Hypothesis: SF negatively relates to adoption (Table 2).

*Slope of the farmland* (*SFL*): A dummy variable (1 for sloped farmland, 0 otherwise). Erosion is more serious on steeper plots. Farmers with sloped plots are more likely to use conservation measures [31]. Hypothesis: Slope positively influences ASWCP (Table 2).

#### 2.11.4. Analysis of Farmers' Perception of Soil Erosion Causes, Extent, and Consequences

A 5-point Likert scale was used to measure the scale of the farmers' perception, and PCA is employed for this purpose. Using PCA with Likert scale data requires special considerations because Likert items are ordinal, not interval. While PCA assumes continuous, normally distributed variables, it can still be applied to Likert data if done carefully, with awareness of the limitations. Understand the nature of Likert data that Likert responses (e.g., 1 = *strongly disagree* to 5 = *strongly agree*) are ordinal, meaning they have a ranked order, but the distances between ranks are not necessarily equal. Standard PCA assumes interval-level measurement, so using raw Likert data directly can lead to biased results. Therefore, the best practice preferably used for this study was polychoric correlations instead of Pearson correlations to better capture the ordinal nature of Likert data.

## 3. Results and Discussions

### 3.1. Descriptive Analysis

#### 3.1.1. Descriptive Analysis of Continuous Variables

The study analyzed the age distribution of respondents (both adopters and nonadopters) in relation to SWC practices. Among adopters, 11.7% fell within the 20–40 age group, while 11.8% of nonadopters were in the same age range. Respondents aged over 60 accounted for 27.9% of adopters and 24.3% of nonadopters. The majority (60.4% of adopters and 63.9% of nonadopters) were between 41 and 60 years old. The average age for the entire sample population was 53.48 years, with a standard deviation of 9.51. Adopters had an average age of 53.69 years, slightly higher than nonadopters (53.25 years). Older farmers are expected to use their farming experience to decide to adopt the SWC technologies. The *t*-test indicated that the age difference between adopters and nonadopters was statistically insignificant (*t* = −0.4027; *p* = 0.3437) (Table 3). In summary, older farmers tended to adopt SWC practices, but the age difference did not significantly impact adoption rates.

Respondents were categorized based on their family size in man equivalent. Small numbers of both adopters and nonadopters fell into the categories of 1–4 members (16.2% and 18.8%, respectively) and ≥ 9 members (11.7% and 7.6%, respectively). The majority of both adopters and nonadopters (72.1% and 73.6%, respectively) had family sizes in the range of five to eight members. The overall average family size in man equivalent for the sample population was 6.25, with a standard deviation of 2.19. Among adopters, the average family size was slightly higher at 6.39, while nonadopters had an average family size of 6.09. Farmers who adopted SWC practices tended to have larger family sizes in man equivalent compared to nonadopters. However, statistical analysis (*t*-test) indicated that the difference in family size between adopters and nonadopters was not significant (*t* = −1.1919; *p* = 0.1171) (Table 4). In summary, family size did not strongly influence the ASWCP in the study area.

The study results indicate that both adopters and nonadopters exhibit varying levels of education. A small percentage of respondents had no formal education or attended primary school (Grades 1–4). Adopters were more likely to have attended secondary school (Grades 9–12) or completed Grades 5–8. The average EDU for adopters was 8.29, while for nonadopters, it was 5.78. The mean EDU for the entire sample population was 7.08. A *t*-test confirmed a significant mean difference between adopters and nonadopters regarding EDU (*p* ≤ 0.001) (Table 5). This finding aligns with previous research [32] showing that higher EDU positively influence the ASWCP. Education plays a crucial role in promoting sustainable practices, empowering farmers with knowledge, and enhancing adoption rates.

The study reveals that both adopters and nonadopters exhibit varying FS. A small percentage of respondents have very large farms (> 3.5 hectares). Adopters are more likely to have FS less than or equal to 1 hectare, while nonadopters tend to have sizes between 1.1 and 3.5 hectares. The average FS for the entire sample population is 1.8 hectares, with a standard deviation of 0.69. Adopters have a slightly larger average FS (1.9 hectares) compared to nonadopters (1.3 hectares). The *t*-test confirms a significant mean difference between adopters and nonadopters regarding FS (*p* ≤ 0.001) (Table 6). This finding aligns with previous research [10] showing that larger land sizes positively influence the adoption of conservation practices. FS plays a crucial role in determining the feasibility and effectiveness of SWC measures.

The study results reveal that both adopters and nonadopters exhibit varying levels of LIO. A small percentage of respondents have very high LIO (> 4 TLUs). Adopters are more likely to have LIO less than or equal to one TLU, while nonadopters tend to have ownership between 1.1 and 5 TLU. The average LIO for the entire sample population is 1.9 TLU, with a standard deviation of 1.24. Adopters have a slightly larger average LIO (2.3 TLU) compared to nonadopters (1.5 TLU). The *t*-test confirms a significant mean difference between adopters and nonadopters regarding LIO (*p* ≤ 0.001) (Table 7). This finding aligns with previous research [28] showing that higher LIO positively influences the ASWCP. LIO plays a crucial role in resource management and sustainable agricultural practices.

Both adopters and nonadopters exhibit varying levels of total income. A small percentage of respondents have very low total income (equal to or less than 15,000 birr). Adopters are more likely to have total income greater than 30,000 birr, while nonadopters tend to fall between 15,001 and 30,000 birr. The average total income for the entire sample population is 25,140.81 birr, with a standard deviation of 10,853.48. Adopters have a slightly higher average total income (26,823.12 birr) compared to nonadopters (23,341.67 birr). The *t*-test confirms a significant mean difference between adopters and nonadopters regarding total income (*p* ≤ 0.01) (Table 8). This finding aligns with previous research [10] showing that higher total income positively influences the ASWCP. Household income plays a crucial role in resource allocation and investment decisions.

Both adopters and nonadopters exhibit varying levels of OFI. A small percentage of respondents have relatively high OFI (greater than 5000 birr). Adopters are more likely to have OFI between 3001 and 5000 birr, while nonadopters tend to fall between 1001 and 5000 birr. The average OFI for the entire sample population is 2794.456 birr, with a standard deviation of 3831.085. Adopters have a slightly lower average OFI (2637.208 birr) compared to nonadopters (2962.625 birr). The *t*-test indicates that there is no significant mean difference between adopters and nonadopters regarding OFI (*p* > 0.05) (Table 9). OFI diversification plays a role in household livelihood strategies and resource allocation.

#### 3.1.2. Descriptive Analysis of Dummy Variables

Among nonadopter households, 49.3% were headed by male nonadopters and 50.7% by female nonadopters. The chi-square test indicates a significant association between the SEX and the ASWCP (*p* ≤ 0.001) (Table 10). This finding aligns with previous research [26], confirming that male-headed households are more likely to adopt SWC practices compared to their female counterparts. Understanding these gender dynamics is essential for promoting sustainable practices and equitable resource management.

Among the respondents, 66.9% with LAO were adopters, while 33.1% were nonadopters. This suggests that LAO plays a crucial role in promoting the adoption of conservation practices. The *χ*^2^ test confirms a significant association between OFI and the ASWCP (*p* ≤ 0.001) (Table 11). This finding aligns with previous research, emphasizing that LAO positively influences farmers' decisions regarding conservation practices. Understanding the impact of LAO can inform policies and interventions to enhance sustainable land management.

Among the respondents, 65.8% with no fertile soil were adopters, while 34.2% were nonadopters. Adopters with no fertile soil significantly outnumbered nonadopters in this category. The chi-square test confirms a significant association between SF and the ASWCP (*p* ≤ 0.001) (Table 12). This finding aligns with previous research [32] emphasizing that SF significantly influences adoption decisions. Understanding soil conditions is crucial for effective conservation planning.

In the context of SWC practices, ES play a crucial role. These services enable households to adopt conservation practices effectively. According to descriptive statistics from a study (referenced as [10]), 74.2% of respondents who had access to ES became adopters of SWC practices, while 25.8% remained nonadopters. The higher adoption rate among those with extension contact can be attributed to improved technical skills, access to agricultural information, and support provided by extension workers. Notably, the proportion of adopters was significantly higher than that of nonadopters. A *χ*^2^ test confirmed a strong association between extension contact and the ASWCP at a significance level (*p* ≤ 0.001) (Table 13). This finding aligns with previous research [33] emphasizing the positive impact of ES on the implementation of conservation practices.

The study found that farmers who received TSWC practices were more likely to adopt these practices. Specifically, 70.3% of respondents with training were adopters, while 29.7% were nonadopters. The *χ*^2^ test revealed a significant association between training and adoption. The proportion of adopters among trained farmers was significantly higher than that of nonadopters. This association was statistically significant at a confidence level (*χ*^2^ = 46.4694; *p* ≤ 0.001) (Table 14). These results align with previous findings [10] that training participation positively influences farmers' decisions regarding SWC practices. Promoting training programs can enhance technical skills and provide valuable agricultural information, encouraging more farmers to adopt sustainable practices.

Among the surveyed respondents, 55.9% of those with sloped farmlands adopted SWC practices, while 44.1% did not. The higher adoption rate among farmers with sloped land can be attributed to the increased vulnerability of such areas to soil erosion. These farmers recognize the importance of conservation practices to mitigate erosion and protect their land. The *χ*^2^ test revealed a significant association between the slope of farmland and the ASWCP at a probability level (*p* ≤ 0.01) (Table 15). This finding aligns with previous research [26] that highlighted the positive impact of slope-related factors on households' tendency to adopt conservation practices.

#### 3.1.3. Descriptive Analysis of Farmers' Perception Toward Causes, Extent, and Consequences of Soil Erosion

The perception of farmers in the study area was ranked based on mean scores:

1. Causes of soil erosion:
  ▪ Damaged conservation structures: Farmers identified this as the primary cause (mean score 4.63).
  ▪ Excessive rainfall: Ranked second (mean score 4.56).
  ▪ Steep slopes: Also considered significant (mean score 4.55).
  ▪ Limited use of SWC practices: Highlighted as a contributing factor (mean score 4.22).
  ▪ Deforestation: Associated with soil erosion (mean score 4.21).
  ▪ Runoff from upslope: Received a lower mean score (2.95).
2. Extent of soil erosion:
  ▪ Severe soil erosion: Ranked highest (mean score 3.15).
  ▪ Moderate soil erosion: Followed (mean score 2.75).
  ▪ No soil erosion: Lower perception (mean score 2.03).
  ▪ Minor soil erosion: Least concern (mean score 1.64).
3. Consequences of soil erosion:
  ▪ Farmland fragmentation: A significant concern (mean score 2.70).
  ▪ Creation of rills and gullies: Also noted (mean score 2.69).
  ▪ Change of crop types: Impacted by erosion (mean score 2.27).
  ▪ Land productivity decline: Substantially reduce crop yields (mean score 1.92).
  ▪ Loss of SF: Recognized (mean score 1.82).

Farmers in the study area prioritize addressing damaged conservation structures, severe soil erosion, and farmland fragmentation. Detailed descriptive analysis of farmers' perceptions toward soil erosion is shown in Table 16.

### 3.2. Implemented SWC Practices in the Study Area

#### 3.2.1. Physical SWC Practices

These practices involve tangible structures and techniques implemented on farmland:

• Soil bund: Soil bunds effectively control soil erosion by retaining rainwater and soil moisture on the farmland. They are constructed to create barriers that prevent runoff.
• Cutoff drain: Cutoff drains help divert excess water away from fields, reducing erosion and waterlogging.
• Check dam: Check dams slow down water flow, allowing sediment to settle and preventing erosion.
• Fanya juu: A conservation practice that stabilizes soil and water flow.
• Contour plow: Tilling the land along the contours of the slope prevents downward runoff on steep terrain. These structures are typically built during the dry season using traditional methods, such as oxen and manual digging.
• Waterways: Designed channels to manage water flow and prevent erosion.

#### 3.2.2. Biological SWC Practices

These methods focus on natural processes and vegetation:

• Planting trees: Trees play a crucial role in stabilizing soil, reducing erosion, enriching soil quality, and maintaining moisture.
• Mixed cropping: By having different crops on the land for longer periods, mixed cropping helps reduce erosion.
• Grass strips: Planting grass strips along contours or slopes helps prevent runoff and soil loss.
• Crop rotation: Rotating crops improves SF, reduces pests and diseases, and minimizes degradation.
• Manure application: Adding organic matter to the soil enhances its structure and nutrient content.
• Following practice: Adhering to sustainable agricultural practices contributes to SWC.

In general, contour plow, soil bund, and cutoff drain are the primary physical conservation practices implemented. Meanwhile, crop rotation, tree planting, and mixed cropping serve as essential biological conservation methods in the study area (Table 17).

### 3.3. Testing of Multicollinearity in the Context of Probit Regression Models

Multicollinearity refers to the presence of strong correlations among independent variables, which can affect the reliability of regression results. Here are the steps taken to address multicollinearity:

1. Variance inflation factor (VIF):
  • VIF assesses the inflation of variance in an estimator due to multicollinearity [33].
  • The formula for VIF is
     $$\begin{array}{c} \text{VIF}\left(\text{Xi}\right)=1-\text{Ri}21 \end{array}$$
    where Ri is the squared multiple correlation coefficient between variable Xi and other explanatory variables [33].
  • Stata statistical tools were used to compute VIF values.
  • A rule of thumb: If the VIF of a variable exceeds 10, there may be a problem of multicollinearity.
  • In this study, VIF values for explanatory variables were found to be small (less than 10), indicating no significant multicollinearity.
  • Therefore, all 13 explanatory variables were retained for the probit regression analysis.
2. Contingency coefficient:
  • The contingency coefficient measures the association between dummy variables using chi-square tests.
  • Values range between 0 and 1: Zero indicates no association, while values close to 1 suggest a high degree of association.
  • After screening the best explanatory variables, no significant association or multicollinearity was detected among the discrete explanatory variables.

Generally, both VIF and contingency coefficient analyses confirmed that multicollinearity was not a problem in this study. Researchers proceeded with the probit regression model using the retained explanatory variables.

Moreover, robust statistical methods were also used as a tool for reducing the sensitivity, through the detection of the outliers by first fitting the majority of the data and then by flagging deviant data points.

### 3.4. Determinants of ASWCP

The probit regression model revealed several determinants that significantly influence households' ASWCP:

*Sex of household head*: The ASWCP was significantly higher (*p* ≤ 0.01) among male-headed households compared to those led by females. This difference is largely attributed to the relatively greater labor availability, larger landholdings, and higher LIO among male-headed households. As a result, male-headed households were found to be 16.89% more likely to adopt SWC interventions than their female counterparts (Table 18). This outcome is consistent with prior research [25], which also reported a greater propensity for SWC adoption among male-headed households. In contrast, a study conducted by [34] in the Koga Watershed of Northern Ethiopia found no association between sex and the adoption of SWC structures, as women in the area are traditionally engaged in farming activities on their own land. These findings underscore the need for gender-responsive policies that address structural disparities in access to resources. Enhancing the role of local institutions and expanding access to education can play a critical role in promoting more inclusive and widespread adoption of sustainable land management practices.

*EDU*: Educational attainment of the household head exhibited a strong and positive influence on the likelihood of adopting SWC practices. Statistically significant at the (*p* ≤ 0.001) level, the analysis revealed that each additional year of formal education increased the probability of adoption by 3.58% (Table 18). Education fosters a better understanding of land degradation issues, enhances awareness of sustainable agricultural practices, and improves decision-making capacity regarding natural resource management. These results are consistent with previous findings [35], highlighting the vital role of education in encouraging the uptake of conservation strategies. Strengthening educational infrastructure and local capacity-building initiatives is essential for fostering wider ASWCP across farming communities in Ethiopia. In contrast, [36] explained that illiterate farmers are more likely to adopt SWC practices compared to educated farmers, who are often occupied with off-farm activities.

*FS*: The analysis revealed a positive relationship between FS and the ASWCP. This variable was statistically significant at the (*p* ≤ 0.001) level in both the robust standard error and marginal effect estimations (Table 18). Households with larger areas of cultivated land were more inclined to implement SWC measures, likely due to the availability of sufficient space and resources necessary for effective conservation interventions. Ample land allows farmers to adopt such practices without compromising their primary agricultural activities. This observation is consistent with prior studies of [34] that have underscored the importance of landholding size in facilitating the adoption of sustainable land management practices. However, [37] presented a contrasting viewpoint.

*ES*: Access to agricultural ES emerged as a key factor positively influencing the uptake of SWC practices. The relationship was significant at the (*p* ≤ 0.001) level, with findings indicating that households receiving extension support were 28.9% more likely to adopt SWC measures compared to those without such access (Table 18). ES offer crucial technical assistance, promote awareness, and provide training on best practices, thereby motivating farmers to engage in conservation efforts. This result is supported by earlier research [34] that highlights the pivotal role of ES in enhancing the adoption of sustainable agricultural technologies.

*TSWC*: Participation in training programs related to SWC practices was found to be the most influential determinant of adoption. Statistically significant at the (*p* ≤ 0.001) level, training was associated with a 34.65% increase in the likelihood of SWC adoption (Table 18). Farmers who had received training were more informed about the adverse effects of soil erosion and the methods available to mitigate it. As a result, they were more capable and willing to apply effective conservation techniques. This outcome aligns with a previous study [36] that has highlighted the transformative impact of targeted training in boosting the adoption of SWC strategies. Thus, providing training and ES serves as a strategy to raise awareness and offer support for the adoption of SWC technologies.

*HTI*: Total household income was also significantly associated with SWC adoption, with results showing significance at the *p* ≤ 0.01 level. An increase in annual household income (measured in Ethiopian birr) corresponded with a greater probability of adopting conservation practices (Table 18). Higher income households have better financial capacity to invest in the materials and labor required to construct and maintain SWC structures. This result aligns with the findings of [34], which indicated that both on-farm and OFI significantly influence a household's likelihood of adopting SWC practices. This reinforces findings from previous literature that point to the enabling role of economic resources in supporting the uptake of land conservation measures.

*SF:* SF was found to significantly (*p* ≤ 0.01) reduce the likelihood of farmers adopting SWC practices. Statistical analysis showed that households with fertile land were 18.15% less likely to implement SWC measures (Table 18). This is likely because farmers perceive fertile soils as already productive and therefore see little immediate benefit in adopting additional conservation strategies. Consequently, the perceived adequacy of soil quality diminishes the perceived benefits or urgency of adopting SWC technologies. These findings highlight a key behavioral barrier: Perceived soil quality can reduce the motivation to invest in long-term land management. Similar trends have been observed in other studies ([38]), reinforcing the need for awareness campaigns and policy incentives that encourage SWC adoption even on fertile lands to maintain productivity over time and prevent future degradation.

On the other hand, variables such as age of household head, HS, LIO, and OFI are not statistically significant in influencing the ASWCP (Table 18).

### 3.5. PCA of Farmers' Perception Toward Soil Erosion

These perceptions were grouped using a PCA whereby related perceptions based on use were grouped into clusters (components). This was important as it enabled subsequent analysis by fitting the groups into the model and reaching conclusions. The approach is superior to the conventional grouping of perceptions, which would make it difficult to conclude about a group in cases where few perceptions could represent the entire group. The components were rotated using orthogonal rotation (Varimax method) [39] so that smaller number of highly correlated perceptions was put under each component for easy interpretation and generalization about a group. The result of the rotation was five principal components (PCs) from a possible 15 extracted with eigenvalues > 1 following the [40] criterion. PCA is useful in reducing the dimensionality of data without loss of much information.  Table 19 contains PCs and the coefficients of linear combinations called loadings. A popular and intuitive index of goodness of fit in multivariate data analysis is the percentage of explained variance: The higher the percentage of variance a proposed model manages to explain, the more valid the model seems to be. Thus, a visual inspection of Table 19 revealed that the five PCs explained 77.54% of total variability in the data set. This presents a good fit indicating that the PCA results highly explained the data. A closer look at each column of Table 19 helps to define each component according to the strongly associated perceptions.

Observed variable (farmer perception) is associated with each underlying component (factor). After rotation, the components are easier to interpret because each variable tends to load highly on just one factor. For the interpretation of the PCs, variables with high factor loadings were considered from the Varimax rotation [41]. Factor loadings (i.e., correlation values in the range [−1, +1]) between each perception measure component (Table 20). In order to construct the factors in this study, the variable loadings that are highly positive (≥ 0.3086) or negative (≤ −0.4103) were loaded strongly with the factor. Further, to define the factors clearly, it was decided to delete variables that had loadings below 0.3086 or greater than −0.4103.

#### 3.5.1. Component 1: Natural and Structural Contributors to Erosion

The first factor (component) accounts for the largest portion of the variation in the original data (26.23%) which are related to causal factors. Variables like slope being steep, too much rainfall, damaged conservation structure, and limited use of SWC practice have loadings of (0.4555), (0.4463), (0.3996), and (0.3086), respectively (Table 20). All these variables had positive loadings. A positive loading indicates the direct relationship between the factor and variable, but magnitude tells the strength of the relationship. In general, Component 1 (one) captures physical and structural causes of erosion. Farmers who load high on this component perceive erosion as a result of topography (slope being steep), climate (too much rainfall), and poor conservation structures.

#### 3.5.2. Component 2: Perceived Presence and Extent of Erosion

The second factor (component) had an eigenvalue of 2.694, and the % of variance was 17.96. This factor entailed four items: moderate soil erosion with loading of 0.3910, minor soil erosion (0.4178), and land productivity decline (0.4414), all with positive loading. This factor can be interpreted as extent and consequence factors. Moreover, this study verifies that farmers' perception of soil erosion is related to extent and consequence factors due to moderate soil erosion, minor soil erosion, and land productivity decline being mirrored through extent and consequence factors. Runoff from upslope has a loading of 0.5655 (Table 20). The factor is interpreted and named as “causal factor.” This factor had an eigenvalue of 2.344 and is estimated to explain 15.63% of the total variance. The variable had a positive loading, which shows a positive association between the variable and the factor. The study further substantiates the relationship between the causal factor and the variable as it plays a significant role in perception because runoff from upslope is imitated through the psychological factor. Hence, the causal factor illustrates that farmers' perception of soil erosion was positive. Finally, the second factor (component) reflects farmers' subjective assessment of erosion on their farms, whether they see it as none, minor, or moderate and whether they link it to runoff or productivity decline.

#### 3.5.3. Component 3: Effects and Outcomes of Erosion

The pattern of factor (component) loading for interpreting three variables, loss of SF, creation of rills and gullies, and farmland fragmentation, has the loadings of (0.3901), (0.5058), and (0.4988), respectively (Table 20). So these variables come under the heading of “consequence factors,” and it is explaining 11.01% of total variance with an eigenvalue of 1.652. The results revealed that loss of SF, creation of rills and gullies, and farmland fragmentation had positive loadings in the original data which implied that there is a positive relation with “consequence factors.” The study further verifies that “consequence factors” play an important role in the perception of farmers toward soil erosion because loss of SF, creation of rills and gullies, and farmland fragmentation all reflected through consequence factors. This factor (component) captures the visible degradation effects of erosion—such as SF loss, physical land damage (gully formation), and fragmentation. It reflects an outcome-based view. This indicates that farmers perceived the consequences of soil erosion.

#### 3.5.4. Component 4: Adaptive Agricultural Responses

The factor loading for interpreting one variable, change of crop types, has the loading (0.3606). The result revealed that change of crop types had positive loadings. This variable comes under the heading of “adaptive agricultural responses.” This single-variable factor (component) shows that some farmers respond to erosion by changing crops, perhaps toward more resilient or low-input varieties. It suggests adaptive behavior.

#### 3.5.5. Component 5: Forest and Erosion Severity Awareness

The factor loadings for interpreting two variables, deforestation and severe soil erosion, have the loadings of (0.3927) and (−0.4103), respectively (Table 20). So these variables come under the heading of “causal, extent, and consequence factors,” and it is explaining 6.71% of total variance with an eigenvalue of 1.007. The results revealed that deforestation had positive loadings in the original data which implied that there is a positive relation with “causal, extent, and consequence factors,” while severe soil erosion had negative loading in the original data which implied that there is a negative relation with causal, extent, and consequence factors. The study further confirms that “causal, extent, and consequence factors” play an important role in the perception of farmers toward severe soil erosion and deforestation. This factor (component) suggests that farmers associate severe erosion with deforestation, though the negative loading on “severe erosion” may indicate denial or underestimation by farmers who cite deforestation as the cause.

In general, the finding of this study revealed that there are five dissimilar components that can play an imperative role behind the farmers' perception of causes, extent, and consequences of soil erosion such as causal factors, extent, and consequence factors.

## 4. Conclusion

The sustainable management of soil and water resources remains a cornerstone of resilient agricultural systems, particularly in erosion-prone areas such as Soro District, Central Ethiopia. This study demonstrates that a range of socioeconomic, institutional, and perceptual factors such as sex, educational attainment, FS, access to ES, participation in SWC training, and overall household income demonstrated a significant and positive impact on the adoption of soil and water management practices, along with farmers' perceptions of erosion severity. Conversely, SF was negatively associated with the adoption of SWC measures. While the majority of farmers generally recognize the negative impacts of soil erosion on land productivity and livelihoods, actual implementation of SWC measures remains inconsistent. To promote sustainable land management and achieve long-term agricultural sustainability in such contexts, policies and interventions must be designed with a clear understanding of local conditions and farmer perspectives. Strengthening rural advisory systems and investing in participatory training programs are essential steps toward improving conservation outcomes. Furthermore, integrating local knowledge and perceptions into program design can foster greater community participation and long-term commitment to conservation practices. The findings of this study contribute to a growing body of evidence highlighting the importance of farmer-centered approaches in sustainable land management. Supporting smallholder farmers through inclusive and adaptive strategies is crucial for safeguarding soil health, enhancing agricultural productivity, and ensuring long-term environmental sustainability in Ethiopia and similar agroecological settings. Future research should explore the long-term effectiveness of SWC interventions and the role of climate variability in shaping land management decisions at the household level.

## Figures and Tables

**Table 1:** Distribution of sample households.

**No.**	**Name of kebele**	**Total household heads**	**Proportion to population size (PPS)**	**Sample size (** **n** **)**		
				**Nonadopter**	**Adopter**	**Total**
1	Bona	320	*n* = (320/1174) × 298	40	41	81
2	First Hankota	391	*n* = (391/1174) × 298	47	52	99
3	Arera	463	*n* = (463/1174) × 298	57	61	118
Total		1174		144	154	298

**Table 2:** Summary of description of independent variables.

**S/N**	**Variable**	**Type of variable measurement**	**Expected sign**
1	Age of the households in years	Continuous	−
2	Sex of the household	Dummy (1 = male, otherwise 0)	+
3	Household size in man equivalent	Continuous	+
4	Education level in years of schooling	Continuous	+
5	Livestock ownership in TLU	Continuous	+
6	Farm size in hectare	Continuous	+
7	Slope of farm land	Dummy (1 if farm land is slope, otherwise 0)	+
8	Land ownership	Dummy (1 if farmland owned by farmer, otherwise 0)	+
9	Extension service	Dummy (1 if farmer got extension service, otherwise 0)	+
10	Training on SWC practices	Dummy (1 if farmer participated training on SWC, otherwise 0)	+
11	Soil fertility	Dummy (1 if fertile farm land, otherwise 0)	−
12	Household total income in birr	Continuous	+
13	Off-farm income in birr	Continuous	−

**Table 3:** Age structure of respondents.

**Age category in years**	**Adopters (** **n** = 154**)**		**Nonadopters (** **n** = 144**)**		**Total (** **n** = 298**)**		**t** ** value (** **p** ** value)**
	**F**	**%**	**F**	**%**	**F**	**%**	
20–40	18	11.7	17	11.8	35	11.7	−0.4027 (0.3437)
41–60	93	60.4	92	63.9	185	62.1	
≥ 60	43	27.9	35	24.3	78	26.2	
Total	154	100	144	100	298	100	

*Note:* Mean age of the adopters = 53.69; SD = 9.53. Average mean = 53.48; SD = 9.51. Mean age of the nonadopters = 53.25; SD = 9.52.

**Table 4:** Distribution of respondents by family size of household.

**Family size in man equivalent (ME)**	**Adopter (** **n** = 154**)**		**Nonadopter (** **n** = 144**)**		**Total (** **n** = 298**)**		**t** ** value (** **p** ** value)**
	**F**	**%**	**F**	**%**	**F**	**%**	
1–4	25	16.2	27	18.8	52	17.5	
5–8	111	72.1	106	73.6	217	72.8	
≥ 9	18	11.7	11	7.6	29	9.7	
Total	154	100	144	100	298	100	−1.1919 (0.1171)

*Note:* Mean household size of the adopters = 6.39 ME; SD = 2.16. Mean household size of the nonadopters = 6.09 ME; SD = 2.21. Average mean = 6.25 ME; SD = 2.19.

**Table 5:** Education level of household head.

**Educational level**	**Adopter (** **n** = 154**)**		**Nonadopter (** **n** = 144**)**		**Total (** **n** = 298**)**		**t** ** value (** **p** ** value)**
	**F**	**%**	**F**	**%**	**F**	**%**	
Unable to read and write	3	1.9	16	11	19	6.4	
Primary school (1–4)	28	18.2	9	6.3	37	12.4	
Primary school (5–8)	38	24.7	97	67.4	135	45.3	
Secondary school (9–12)	85	55.2	22	15.3	107	35.9	
Total	154	100	144	100	298	100	−7.3803 (0.001)⁣^∗∗∗^

*Note:* Mean education level of the adopters = 8.29; SD = 2.69. Mean education level of the nonadopters = 5.78; SD = 3.18. Average mean = 7.0; SD = 3.19.

⁣^∗∗∗^ statistically significant at 0.001 likelihood level.

**Table 6:** Farm size of sample households.

**Farm size in hectare**	**Adopter (** **n** = 154**)**		**Nonadopter (** **n** = 144**)**		**Total (** **n** = 298**)**		**t** ** value (** **p** ** value)**
	**F**	**%**	**F**	**%**	**F**	**%**	
≤ 1	23	14.9	78	54.2	101	33.9	
1.1–3.5	130	84.4	64	44.4	194	65.1	
> 3.5	1	0.7	2	1.4	3	1	
Total	154	100	144	100	298	100	−7.8431 (0.001)⁣^∗∗∗^

*Note:* Mean farm size of the adopters = 1.9; SD = 0.59. Mean farm size of the nonadopters = 1.3; SD = 0.67. Average mean = 1.8; SD = 0.69.

⁣^∗∗∗^ statistically significant at 0.001 likelihood level.

**Table 7:** Livestock ownership.

**Livestock ownership in TLU**	**Adopter (** **n** = 154**)**		**Nonadopter (** **n** = 144**)**		**Total (** **n** = 298**)**		**t** ** value (** **p** ** value)**
	**F**	**%**	**F**	**%**	**F**	**%**	
≤ 1	42	27.3	86	59.7	128	42.9	
1.1–4	103	66.9	54	37.5	157	52.7	
Above 4	9	5.8	4	2.8	13	4.4	
Total	154	100	144	100	298	100	−5.6392 (0.001)⁣^∗∗∗^

*Note:* Mean livestock of the adopters = 2.3 TLU; SD = 1.22. Mean livestock of the nonadopters = 1.5 TLU; SD = 1.14. Average mean = 1.9 TLU; SD = 1.24.

⁣^∗∗∗^ statistically significant at 0.001 likelihood level.

**Table 8:** Household total income distribution percent by households.

**Total income in birr**	**Adopter (** **n** = 154**)**		**Nonadopter (** **n** = 144**)**		**Total (** **n** = 298**)**		**t** ** value (** **p** ** value)**
	**F**	**%**	**F**	**%**	**F**	**%**	
≤ 15,000	21	13.6	34	23.6	55	18.5	−2.7987 (0.0027)⁣^∗∗∗^
15,001–30,000	81	52.6	82	57	163	54.7	
> 30,000	52	33.8	28	19.4	80	26.8	
Total	154	100	144	100	298	100	

*Note:* Mean total income of the adopters = 26,823.12 birr; SD = 10,850.36. Mean total income of the nonadopters = 23,341.67 birr; SD = 10,601.28. Average mean = 25,140.81 birr; SD = 10,853.48.

⁣^∗∗∗^ statistically significant at 0.001 likelihood level.

**Table 9:** Off-farm income distribution percent by households.

**Off-farm income in birr**	**Adopter (** **n** = 154**)**		**Nonadopter (** **n** = 144**)**		**Total (** **n** = 298**)**		**t** ** value (** **p** ** value)**
	**F**	**%**	**F**	**%**	**F**	**%**	
≤ 3000	120	77.9	112	77.7	232	77.9	0.7322 (0.2323)
3001–5000	10	6.5	9	6.3	19	6.4	
> 5000	24	15.6	23	16	47	15.7	
Total	154	100	144	100	298	100	

*Note:* Mean off-farm income of the adopters = 2637.208 birr; SD = 3484.865. Mean off-farm income of the nonadopters = 2962.625 birr; SD = 4175.5. Average mean = 2794.456 birr; SD = 3831.085.

**Table 10:** Sex of sample household head.

**Description**	**Adopter (** **n** = 154**)**		**Nonadopter (** **n** = 144**)**		**Total (** **n** = 298**)**		**χ** ^2^ ** value (** **p** ** value)**
	**F**	**%**	**F**	**%**	**F**	**%**	
Male	114	74	71	49.3	185	62.1	19.3179 (0.001)⁣^∗∗∗^
Female	40	26	73	50.7	113	37.9	
Total	154	100	144	100	298	100	

⁣^∗∗∗^ is significant at 1% likelihood level separately.

**Table 11:** Land ownership.

**Household response**	**Adopter (** **n** = 167**)**		**Nonadopter (** **n** = 129**)**		**Total (** **n** = 296**)**		**χ** ^2^ ** value (** **p** ** value)**
	**F**	**%**	**F**	**%**	**F**	**%**	
Yes	101	68.3	50	27.1	151	50.3	28.3569 (0.001)⁣^∗∗∗^
No	53	31.7	94	72.9	147	49.7	
Total	154	100	144	100	298	100	

⁣^∗∗∗^ statistically significant at 0.001 likelihood level.

**Table 12:** Soil fertility.

**Household response**	**Adopter (** **n** = 154**)**		**Nonadopter (** **n** = 144**)**		**Total (** **n** = 298**)**		**χ** ^2^ ** value (** **p** ** value)**
	**F**	**%**	**F**	**%**	**F**	**%**	
Yes	58	37.7	94	65.3	152	51	22.7095 (0.001)⁣^∗∗∗^
No	96	62.3	50	34.7	146	49	
Total	154	100	144	100	298	100	

⁣^∗∗∗^ statistically significant at 0.001 likelihood level.

**Table 13:** Extension service.

**Household response**	**Adopter (** **n** = 154**)**		**Nonadopter (** **n** = 124**)**		**Total (** **n** = 294**)**		**χ** ^2^ ** value (** **p** ** value)**
	**F**	**%**	**F**	**%**	**F**	**%**	
Yes	112	72.7	39	27.1	151	50.7	62.0257 (0.001)⁣^∗∗∗^
No	42	27.3	105	72.9	147	49.3	
Total	154	100	144	100	298	100	

⁣^∗∗∗^ statistically significant at 0.001 likelihood level.

**Table 14:** Training on soil and water conservation practices.

**Household response**	**Adopter (** **n** = 154**)**		**Nonadopter (** **n** = 144**)**		**Total (** **n** = 298**)**		**χ** ^2^ ** value (** **p** ** value)**
	**F**	**%**	**F**	**%**	**F**	**%**	
Yes	111	72.1	47	32.6	158	53	46.4694 (0.001)⁣^∗∗∗^
No	43	27.9	97	67.4	140	47	
Total	154	100	144	100	298	100	

⁣^∗∗∗^ statistically significant at 0.001 likelihood level.

**Table 15:** Slope of farmland.

**Household response**	**Adopter (** **n** = 154**)**		**Nonadopter (** **n** = 144**)**		**Total (** **n** = 298**)**		**χ** ^2^ ** value (** **p** ** value)**
	**F**	**%**	**F**	**%**	**F**	**%**	
Flat (≤ 5%)	22	14.3	40	27.8	62	20.8	8.2215 (0.004)⁣^∗∗∗^
Slope (> 5%)	132	85.7	104	72.2	236	79.2	
Total	154	100	144	100	298	100	

⁣^∗∗∗^ statistically significant at 0.001 likelihood level.

**Table 16:** Descriptive analysis of farmers' perception on soil erosion.

**Perception**	**Mean**	**Std. Dev.**	**Rank**
Deforestation	4.214765	0.761566	
Slope being steep	4.550336	0.790825	3rd
Too much rainfall	4.560403	0.794367	2nd
Damaged conservation structures	4.630872	0.785808	1st
Limited use of SWC practices	4.218121	0.97584	4th
Runoff from upslope	2.95302	1.776523	6th
Moderate soil erosion	2.748322	1.397598	2nd
Severe soil erosion	3.151007	1.318356	1st
Minor soil erosion	1.637584	1.049064	4rd
No soil erosion	2.033557	0.670857	3rd
Change of crop types	2.271812	1.478218	3rd
Loss of soil fertility	1.818792	0.72987	5th
Land productivity decline	1.916107	1.181939	4th
Creation of rills and gullies	2.697987	1.386344	2nd
Farmland fragmentation	2.704698	1.390218	1st

**Table 17:** Major SWC practices implemented in the study area among rural households.

	**Frequency**	**%**	**Rank**
Physical (structural) practices			
Soil bund	105	35.2	2nd
Cutoff drain	42	31.9	3rd
Check dam	36	14.4	6th
Fanya juu	80	26.8	4th
Contour plow	110	36.9	1st
Waterways	65	21.8	5th
Biological SWC practices			
Planting of trees	52	17.5	2nd
Mixed cropping	40	13.4	3rd
Grass strips	38	12.8	4th
Crop rotation	78	26.2	1st
Manure application	21	7	5th
Fallowing practice	6	2	6th

**Table 18:** Probit regression model for determinants of adoption of SWC practices.

**Probit regression**		**Number of obs = 298**			
		**Wald chi^2^ 13 = 116 57**			
		**Prob > chi^2^ = 0 0000**			

**Log pseudolikelihood = −123 03678**					

**Variable**	**Coef.**	**Pseudo** **R** ^2^ = 0.4039			
		**DY/DX**	**Robust Std. Err.**	**Z**	**p** > **z**

Age of HH head	0.011075	0.004415	0.011366	0.97	0.33
Sex of HH head⁣^∗∗^	0.426595	0.168886	0.1838	2.32	0.01
Household size	0.042918	0.017111	0.044433	0.97	0.334
Education level⁣^∗∗∗^	0.089694	0.035759	0.030682	2.92	0.001
Farm size⁣^∗∗∗^	0.547583	0.218311	0.18391	2.98	0.001
Extension service⁣^∗∗∗^	0.741459	0.289026	0.203651	3.64	0.001
Land ownership	0.11847	0.047206	0.190952	0.62	0.535
Slope of farmland	0.161183	0.064229	0.212825	0.76	0.449
Training on SWC⁣^∗∗∗^	0.89781	0.346486	0.184869	4.86	0.001
Soil fertility⁣^∗∗^	−0.45939	−0.18152	0.188974	−2.43	0.01
Livestock ownership	0.082949	0.03307	0.089544	0.93	0.354
Off-farm income	−3.3e − 05	−1.3e − 05	2.48e − 05	−1.32	0.186
HH total income⁣^∗∗^	2.15e − 05	8.58e − 06	8.60e − 06	2.5	0.01
_cons	−3.99899		0.798327	−5.01	0.001

⁣^∗∗∗^ and ⁣^∗∗^ statistically significant at 0.001 and 0.01 likelihood levels separately.

**Table 19:** Total variance explained after extraction for farmers' perception toward soil erosion.

**Variable**	**Total variance explained**					
	**Initial eigenvalues**			**Extraction sums of squared loadings**		
	**Total**	**% of variance**	**Cumulative %**	**Total**	**% of variance**	**Cumulative %**
1	3.935	26.23	26.23	3.935	26.23	26.23
2	2.694	17.96	44.19	2.694	17.96	44.19
3	2.344	15.63	59.82	2.344	15.63	59.82
4	1.652	11.01	70.83	1.652	11.01	70.83
5	1.007	6.71	77.54	1.007	6.71	77.54
6	0.929	6.19	83.74			
7	0.581	3.87	87.61			
8	0.473	3.16	90.76			
9	0.382	2.54	93.31			
10	0.323	2.15	95.46			
11	0.244	1.63	97.09			
12	0.183	1.22	98.31			
13	0.129	0.86	99.17			
14	0.113	0.75	99.93			
15	0.011	0.07	100			

*Note:* Extraction method: principal component analysis.

**Table 20:** Rotated component matrix for farmers' perception toward soil erosion.

**Variables**	**Rotated component matrix**				
	**Components (C)**				
	**C1**	**C2**	**C3**	**C4**	**C5**
Deforestation					0.3927
Slope being steep	0.4555				
Too much rainfall	0.4463				
Damaged conservation structure	0.3996				
Limited use of soil and water conservation practice	0.3086				
Runoff from upslope			0.5655		
Severe soil erosion					−0.4103
Moderate soil erosion		0.3910			
Minor soil erosion		0.4178			
No soil erosion					0.5121
Change of crop types					0.3606
Loss of soil fertility				0.3901	
Land productivity decline		0.4414			
Creation of rills and gullies				0.5058	
Farmland fragmentation				0.4988	

*Note:* Extraction method: principal component analysis. Rotation method: Varimax with Kaiser normalization.

## Data Availability

Data will be available on reasonable request from the authors.
